# Industry 4.0 Technologies in Maternal Health Care: Bibliometric Analysis and Research Agenda

**DOI:** 10.2196/47848

**Published:** 2024-08-08

**Authors:** Khulekani Sibanda, Patrick Ndayizigamiye, Hossana Twinomurinzi

**Affiliations:** 1 Department of Applied Information Systems University of Johannesburg Johannesburg South Africa; 2 Centre for Applied Data Science University of Johannesburg Johannesburg South Africa

**Keywords:** Industry 4.0, Fourth Industrial Revolution, bibliometrics, maternal health care, antenatal care, postnatal care, lean operations, maternal, pregnancy monitoring, pregnancy, maternal care, monitoring, thematic analysis, data mining, machine learning, IoT, Internet of Things, artificial intelligence, deep learning, digital health, wearable, mobile phone

## Abstract

**Background:**

Industry 4.0 (I4.0) technologies have improved operations in health care facilities by optimizing processes, leading to efficient systems and tools to assist health care personnel and patients.

**Objective:**

This study investigates the current implementation and impact of I4.0 technologies within maternal health care, explicitly focusing on transforming care processes, treatment methods, and automated pregnancy monitoring. Additionally, it conducts a thematic landscape mapping, offering a nuanced understanding of this emerging field. Building on this analysis, a future research agenda is proposed, highlighting critical areas for future investigations.

**Methods:**

A bibliometric analysis of publications retrieved from the Scopus database was conducted to examine how the research into I4.0 technologies in maternal health care evolved from 1985 to 2022. A search strategy was used to screen the eligible publications using the abstract and full-text reading. The most productive and influential journals; authors’, institutions’, and countries’ influence on maternal health care; and current trends and thematic evolution were computed using the *Bibliometrix* R package (R Core Team).

**Results:**

A total of 1003 unique papers in English were retrieved using the search string, and 136 papers were retained after the inclusion and exclusion criteria were implemented, covering 37 years from 1985 to 2022. The annual growth rate of publications was 9.53%, with 88.9% (n=121) of the publications observed in 2016-2022. In the thematic analysis, 4 clusters were identified—artificial neural networks, data mining, machine learning, and the Internet of Things. Artificial intelligence, deep learning, risk prediction, digital health, telemedicine, wearable devices, mobile health care, and cloud computing remained the dominant research themes in 2016-2022.

**Conclusions:**

This bibliometric analysis reviews the state of the art in the evolution and structure of I4.0 technologies in maternal health care and how they may be used to optimize the operational processes. A conceptual framework with 4 performance factors—risk prediction, hospital care, health record management, and self-care—is suggested for process improvement. a research agenda is also proposed for governance, adoption, infrastructure, privacy, and security.

## Introduction

The Fourth Industrial Revolution, often referred to as Industry 4.0 (I4.0), has revolutionized all sectors with smart technology [1]. For example, Dolgui and Ivanov [2] ⁠posit that I4.0 technologies improve efficiency in manufacturing with minimal use of resources. The increased adoption of I4.0 technologies drives the cyber-physical transformation of manufacturing, logistics, and supply chain in business. There has been a heightened interest in adopting and adapting I4.0 technologies in health care delivery [3]. Li and Carayon [4] point out that using I4.0 tools has transformed the provision of digital health services, such as electronic health records, wearable devices, and storage, among other tools, and has led to improved health outcomes and quality of care. Implementing I4.0 in health care has led to a new term being coined, “Health 4.0,” which is a deployment of health care driven by the success of I4.0 [5]. Maternal health care is one aspect that can benefit from increased efficiencies and quality of care. According to Ahsan et al [6], several health professionals are using new technologies to monitor and improve the quality of patient care they provide remotely. For example, the condition of the mother and the fetus can be monitored using nonintrusive remote monitoring tools [7,8]. The literature on I4.0 is expanding exponentially, with each successive evolution characterized by an increase in the development and innovation of new tools to facilitate how patients and service providers interact. Sustainable Development Goal (SDG) 3.1 [9] aims to ensure access to health care facilities and reduce the global maternal mortality ratio to less than 70 per 100,000 live births. The challenge faced by society and organizations is determining how best they can take advantage of I4.0 technology to ensure the success of SDG 3.1.

While previous research has explored the general potential of I4.0 technologies for health care, specific applications in maternal health care remain underexplored. Luo et al [10] offer valuable insights through a bibliometric analysis but lack a deeper exploration of real-world impacts on practices, policies, and patient outcomes. Similarly, Guo et al [11] highlight the growth of artificial intelligence (AI) research in health care but fail to delve into specific applications with the most significant potential for improvement. Existing reviews on I4.0 technologies [5,6,12] offer broad overviews of applications and management but lack specific use cases and their long-term societal and health care system impacts. This study addresses this gap by investigating the challenges and successes of implementing I4.0 technologies in various maternal health care settings. A conceptual framework for implementing I4.0 technologies in maternal health care is proposed. Further, we highlight insights and directions by mapping the thematic landscape of I4.0 in maternal health care. A research agenda is also developed for future research direction. A total of 136 papers were analyzed bibliometrically. The objectives were to (1) determine the most productive and influential journals in I4.0 research in maternal health; (2) determine authors’, institutions’, and countries’ influence on I4.0 research in maternal health; (3) investigate the current trends and thematic evolution of I4.0 research in maternal health; and (4) develop a conceptual framework for guiding the implementation of I4.0 technologies in maternal health.

In addition, to gain further insight into the use of I4.0 technologies in health care, three research questions were explored: (1) What are the most critical applications of I4.0 in maternal health care? (2) To what extent is I4.0 technology used in maternal health care? and (3) What are the open research issues and areas for future research?

## Methods

### Search and Screening Strategy

The first step was to search for relevant research. The search was conducted on the Scopus electronic database because it indexes peer-reviewed research used by multidisciplinary researchers. Only articles in English (journals, conference papers, and book chapters) were reviewed to focus the study. According to Paul et al [12], I4.0 applications, the Internet of Things (IoT), big data analytics (BDA), cloud computing, blockchain, and AI have had a significant impact on Health 4.0. A search string was thus formulated to focus only on these I4.0 technologies (applications). The primary focus was also limited to maternal health care. The following search string was thus formulated: *((“IoT” OR “Internet of Things”) OR (Big Data Analytics) OR (“Cloud Computing” OR “Cloud”) OR (“Blockchain” OR “Block chain”) OR (“Artificial Intelligence” OR “AI”)) AND (maternal)).*

**Figure 1:**
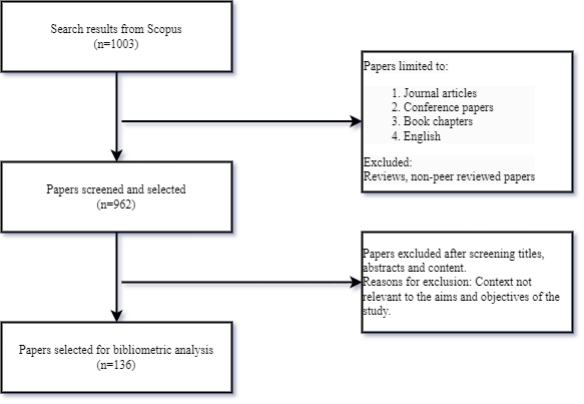
Flowchart summarizing the retrieval strategies.

A total of 1003 unique papers in English were retrieved using the search string, and 136 papers were retained after implementation of the inclusion and exclusion criteria, covering 37 years from 1985 to 2022. Our search strategy encompassed all published scholarly literature on the application of IoT, AI, BDA, cloud computing, and blockchain technologies in the domain of maternal health care. We included journal articles, conference proceedings, and relevant book chapters. To ensure the quality and focus of the analysis, we restricted our selection to (1) papers published in the English language and (2) those directly addressing I4.0 technologies and maternal health care. We excluded from the analysis editorials, news articles, discussion comments, and review papers; articles that discuss maternal health care but does not mention I4.0 technologies; and articles that highlight I4.0 technologies but merely mention maternal health care. Figure 1 illustrates the retrieval strategy.

A total of 623 authors contributed to the literature between 1985 and 2022. The papers were mainly cowritten, with an average of 5.22 authors per paper. All the papers were multiauthored, save for 1 paper. The earliest publication was by Hernandez et al [13]⁠, entitled “Expert system for prediction of foetal condition during labour,” presented at the *IEEE-Engineering in Medicine and Biology Society Annual Conference* in 1985. The latest published paper by Jaba Deva Krupa et al [14], ⁠entitled “An IoMT enabled deep learning framework for automatic detection of foetal QRS: A solution to remote prenatal care,” was published in the *Journal of King Saud University - Computer and Information Sciences*.

### Method of Analysis

Bibliometric techniques were used to analyze the development of I4.0 technologies in maternal health care. Bibliometrics research has been used extensively to map the development of research, including lean supply chain management [15], traceability and lifecycle relationship [16], health technology research [10], supply chain management [17], AI in health care [11], and the theory of constraints [18]. According to Cobo et al [19] and Noyons et al [20], bibliometric analysis has 2 main objectives: research mapping analysis and academic performance analysis. Science mapping as a bibliometric technique helps monitor the structure and evolution of research by reviewing relationships among authors, disciplines, and areas of study [21]. Performance analysis quantitatively and qualitatively measures an entire field of research to determine the most effective and productive research. Further, Cobo et al [19] state that while most research focuses on measuring the scientific performance of a group of actors (countries, universities, departments, and researchers), there is a need to measure the performance of given research conceptually by interrogating specific themes or whole thematic areas.

**Figure 2:**
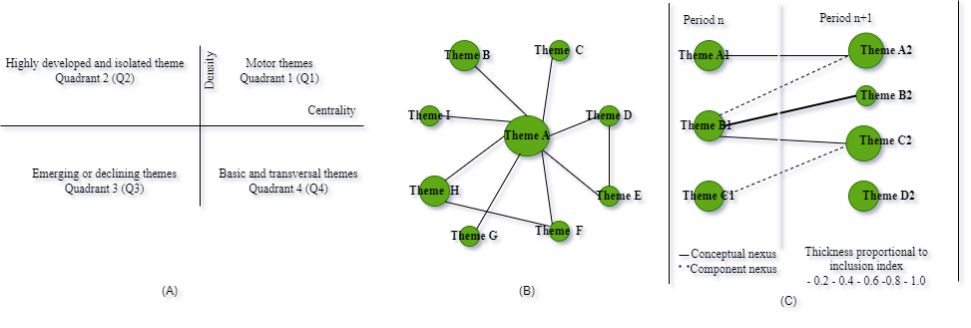
(A) Strategic diagram, (B) thematic network, and (C) thematic evolution [23].

The *Bibliometrix* R package [22] was used to compute the descriptive statistics and bibliometric analysis, including the articles’ analysis, based on the year of publication, citations, authors, affiliation, keywords, coauthorship, and thematic evolution. The conceptual science mapping was conducted following the 4-step methodology proposed by Cobo et al [19]. The first step is to detect research themes. For each period, keywords are extracted from the identified papers to build a network based on a keyword co-occurrence. The nodes represent the keywords, while an edge connects 2 nodes if they coappear in a set of papers [15]. Similar items constitute a cluster and are calculated based on the frequency of keyword co-occurrences. The second step is to visualize identified themes and thematic networks. A graphic representation of the detected themes is presented in a strategic diagram (Figure 2) and a thematic network [23]. Two dimensions are used to characterize each theme—centrality and density. Centrality measures the degree of interaction of the network with other networks, while density is the internal strength of the network [24]. According to Cobo et al [19], the themes can be classified into four quadrants: (1) motor themes (quadrant 1): they exhibit strong centrality and high density; themes in this quadrant are considered well-developed and suitable for structuring a research field; (2) niche themes (quadrant 2): these are highly developed and isolated themes considered of marginal importance to the research field; (3) emerging or declining themes (quadrant 3): these themes exhibit low density and centrality; thus, they may be considered emerging or disappearing; and (4) basic themes (quadrant 4): these represent relevant but not well-developed themes. The third step is to analyze the evolution of detected themes. The research themes are analyzed using an evolution map. The last step is to carry out the performance analysis. The production and scientific impact is measured quantitatively and qualitatively.

## Results

### Overview

This section describes the evolution of I4.0 in maternal health care in terms of publications, citations, and impact by analyzing the following bibliometric indicators—published articles, most productive and influential journals, most productive and influential authors, and most productive and influential institutions and countries. The bibliometric performance analysis is structured into 2 parts—the production and impact of papers and the impact of authors, journals, countries, and research areas.

### Publications and Citations

**Figure 3:**
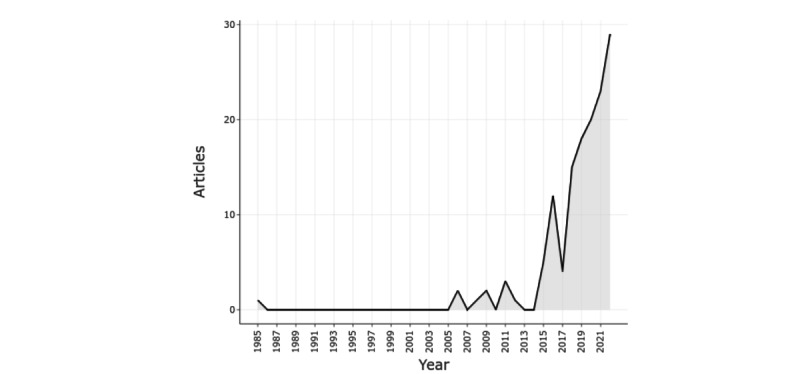
Distribution of publications (1985-2022).

Figure 3 shows the distribution of publications per year and the number of publications (NP) has increased in recent years. Three periods can be observed in the development of publications—1985-2005, 2006-2015, and 2016-2022. The initial period spans 20 years, with only 1 publication in 1985 indicating that I4.0 technologies still needed to be fully adopted in maternal health care. The second period saw 14 publications. However, it can be noted that there was a fluctuation in terms of publications. There was an exponential growth in publications in the last period, with 121 articles published (121/136, 88.9% of all publications in 1985-2022). The annual growth rate of publications for this period is 15.84% compared to the overall growth rate, which stands at 9.53%, implying an increasing NP.

**Figure 4:**
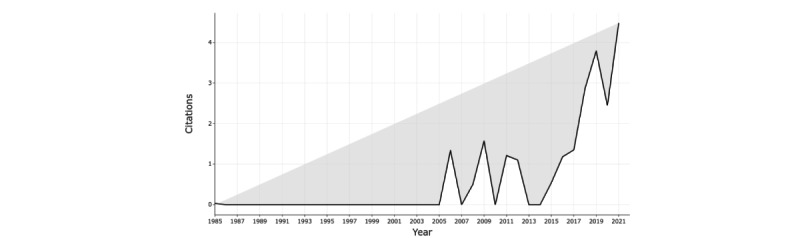
Citations by year (1985-2022).

Figure 4 shows the average citations per year. The trends mirror the publications per year with no citations before 2005. However, from 2006 to 2015, there were fluctuations in citations, with the highest average (1.59, SD 0.40) for the period recorded in 2009. In the last period, there was a gradual increase in the number of citations from a low of 0.5 to a high of 4.5 per year. The increase in research may be traced back to 2011 when the term “Industry 4.0” was first coined [10]. Given their identified advantages, there was a deliberate effort to increase and adopt I4.0 technologies [25]. The slow and sporadic adoption within health care can be attributed to several factors; extensive capital investments were required to implement these technologies and train the workforce, thus averting job disruption risks [26]. Studies also underscore limitations like a lack of adequate infrastructure, a shortage of digital skills among the workforce, the risk of security breaches, the potential disruption of existing jobs, and challenges in ensuring consistent data quality as significant barriers to I4.0 integration [27,28].

### Most Productive Authors and Authors Impact

Table 1 shows the most productive authors during the 1985-2022 period. It gives the NP of the first 10 productive researchers. Moreira and Rodrigues have produced the highest NP with 8 each. They are followed by 4 other researchers with 5 publications each. These results indicate that the research on implementing I4.0 technology is widespread, with no one dominant researcher in the field. Table 1 also shows the most relevant authors and their local impact. It quantifies the contribution of an individual author to a published set of articles. The publication start year indicates the first year the author was published.

**Table 1:** Top 10 most relevant authors and author’s local impact.

Authors	NP^a^	h_index	g_index	m_index	TC^b^	PY_start^c^
Moreira	8	6	8	0.857	122	2016
Rodrigues	8	6	8	0.857	122	2016
Axelin	5	4	5	1.000	85	2019
Azimi	5	4	5	1.000	85	2019
Liljeberg	5	4	5	1.000	85	2019
Rahmani	5	4	5	1.000	85	2019
Saleem	4	4	4	0.571	86	2016
Abelha	3	2	2	0.250	7	2015
Kumar	3	3	3	0.600	67	2018
Machado	3	2	2	0.250	7	2015

^a^NP: number of publications.

^b^TC: total number of citations.

^c^PY_start: the year of first publication of articles.

### Top 10 Most Local Cited Authors

Local cited authors measure how many times the authors have cited an author included in this corpus. Accordingly, this can be interpreted as a measure of the impact of authors conducting research on I4.0 in maternal health care. Moreira and Rodrigues, authors with more scientific production and the highest impact, are cited more (4 times) by other authors in the corpus. It should be noted that the low number of citations may be attributed to the fact that the core technologies behind I4.0 in maternal health care are still in development and are yet to gain wider traction and acceptance [3,12].

### Most Local Cited Sources, Most Relevant Sources, Source Local Impact, and Source Dynamics

The local citations are taken from the reference lists and measure the number of times other papers in the corpus cite a paper. The paper has to appear in at least 1 of the reference lists of the other papers in the collection. In our collection, we have 2244 cited sources. *Fertility and Sterility* stands out with 63 articles, followed by *American Journal of Obstetrics & Gynecology* with 50 articles, *Reproductive Medicine and Biology* with 33 articles, *PLOS ONE* with 32, *Human Reproduction* with 31 articles, and with *BMC Pregnancy and Childbirth* at number 5 with 25 articles.

The most relevant source is the *Lecture Notes in Computer Science* (including subseries *Lecture Notes in Artificial Intelligence* and *Lecture Notes in Bioinformatics*), with 4 publications and a total citation count of 10. The *Journal of Maternal-Fetal & Neonatal Medicine* has the most citations with a total of 44 citations. However, with 3 publications, it is the third most relevant source.

### Most Relevant Affiliations

The most relevant affiliation is the University of Turku in Finland with 11 articles, followed by Brazilian universities, namely, the University of Fortaleza (second with 7 articles), the National Institute of Telecommunications (fifth with 5 articles), and the Instituto Federal De Educação (eighth with 4 articles). The University of California and the University of California, Irvine in the United States are tied for third, with 6 articles each. The rest of the institutions are from Asia, accounting for 4 of the top 10 relevant affiliations—Shenzhen Technology University (sixth with 5 articles) and Anna University, Jinan University, and King Saud University, with 4 articles each.

### Country Scientific Production and Most Cited Countries

The United States is positioned in the first place with a frequency of 80; followed by India with a frequency of 50; and China, Brazil, and Portugal making up the top 5 with a frequency of 34, 30, and 17, respectively. The top 10 most prolific countries are thus located in the United States, Asia, and Europe. However, it can be noted that some research focusing on I4.0 technologies in health care emanates from Africa. Most of this research is concentrated in East Africa—Kenya (n=5), Tanzania (n=2), and Uganda (n=2)—with a frequency of 9 combined. Nigeria is the most prolific country in Africa, with a frequency of 8. South Africa has a frequency of 2.

The most cited countries represent the origin of scientific production referenced by the authors from the collection. Most of the articles reference articles with their origin—Finland (77 citations); Canada (67 citations); the United States (61 citations); and China and Portugal, with 60 citations each.

### Most Global and Local Cited Papers

Table 2 shows the 10 most relevant studies sorted by total citations. A globally cited paper is an article in the collection that has been cited by other papers indexed by a particular database, which in this study was Scopus. The total number of citations represents the total citations received by a selected article “all over the world.” The most frequently cited work was “Missing data resilient decision-making for health care IoT through personalisation: A case study on maternal health” [29]. The objective was to develop a personalized missing data resilient system in an IoT monitoring system, as data acquisition is generally interrupted in long-term monitoring systems. The paper has 55 citations and averages 13.75 per year.

**Table 2:** Top 10 most globally cited papers.

Paper	TC^a^	TC/y^b^
Azimi et al [30]	55	13.75
Catley et al [31]	41	2.41
Moreira et al [29]	36	9.00
Akbulut et al [32]	36	7.20
Rigla et al [33]	35	7.00
Sayers et al [34]	31	2.21
Kumar et al [35]	22	11.0
Moreira et al [36]	22	4.40
Miyagi et al [37]	21	5.25
Tejera et al [38]	21	1.75

^a^TC: total number of citations.

^b^TC/y: total number of citations per year.

The leading papers on I4.0 in maternal health care are presented in Table 3. Only the first 10 top publications are presented based on the local citations. All the papers were published within the last 5 years (2017-2021). The most cited paper is by Azimi et al [30], which is also the most globally cited publication.

**Table 3:** Top 10 most influential papers on I4.0 in maternal health care.

Authors	Title	Summary of research	Source	LC^a^	GC^b^
Azimi et al [30]	Missing data resilient decision-making for health care IoT through personalisation: a case study on maternal health	This research proposed an approach that considers variability and context information to minimize bias when inputting missing data values.	FGCS^c^	3	55
Miyagi et al [39]	Feasibility of deep learning for predicting live birth from a blastocyst image in patients classified by age	An AI^d^ classifier using deep learning with convolutional neural networks (CNN) was developed. The model used images of blastocysts categorized by maternal age to predict the likelihood of achieving a live birth. The authors argue that the model provides an efficient, quick, and economical diagnosis means and permits remote examination.	RMB^e^	2	21
Lopez et al [40]	Wearable technology model to control and monitor hypertension during pregnancy	A technological model was proposed to monitor the care of pregnant women through alerts provided by a group of health parameters. The model combines a group of the patients’ parameters, such as blood pressure, heart rate, and physical technology with a wearable device.	CISTI^f^	2	15
Yarlapati et al [41]	Early prediction of LBW cases via minimum error rate classifier: a statistical machine learning approach	The authors reformulated a forecasting problem as a classification problem between low birth weight (LBW) and not low birth weight (NOT-LBW). They implemented a model using health indicators of pregnant women for early detection of potential LBW.	ICSC^g^	2	8
Moreira et al [42]	Smart mobile system for pregnancy care using body sensors	Presents a mobile monitoring solution to indicate high-risk pregnant women enduring hypertension. The mobile system alerts health care staff if there is a change in hypertension condition during pregnancy.	ICST^h^	2	20
Ueno et al [43]	Pregnancy prediction performance of an annotation-free embryo scoring system on the basis of deep learning after single vitrified-warmed blastocyst transfer: a single-center large cohort retrospective study	The authors discussed an embryo assessment model, iDAScore, developed using deep learning. The model performed better than an annotation-dependent ranking tool or traditional embryo assessment tools.	FAS^i^	1	7
Li et al [44]	The impact of healthcare monitoring technologies for better pregnancy	The authors performed an experimental analysis based on a sample of 315 pregnant women. The study highlighted a high recognition and acceptance of wearing wearable Internet of Things (IoT) devices during pregnancy by pregnant women.	ICET^j^	1	2
Huang et al [45]	Using deep learning in a monocentric study to characterise maternal immune environment for predicting pregnancy outcomes in the recurrent reproductive failure patients	The researchers applied artificial intelligence to analyze patients’ medical information with recurrent reproduction failure (RRF). A machine learning model to predict the pregnancy outcomes for patients with RRF at any gestational period, namely, biochemical pregnancy, clinical pregnancy, ongoing pregnancy, and live birth was developed	FII^k^	1	5
Sarhaddi et al [46]	Long-term IoT-based maternal monitoring: System design and evaluation	An IoT-based system to monitor maternal health during pregnancy and postpartum was developed. Further, an artificial intelligence method for analyzing the data was integrated. The researchers implemented a proof-of-concept monitoring system for an actual human participant study.	SSS^l^	1	5
Mhajna et al [47]	Wireless, remote solution for home fetal and maternal heart rate monitoring	The researchers developed a solution to fuse information gathered from a wireless abdominal belt that is self-applied to obtain fetal heart rate (FHR) and maternal heart rate (MHR).	AJOG^m^	1	17

^a^LC: local citations.

^b^GC: global citations.

cFGCS: Future Generation Computer Systems.

^d^AI: artificial intelligence.

^e^RBM: Reproductive Medicine and Biology.

^f^CISTI: Conference on Information Systems and Technologies.

^g^ICSC: 2017 IEEE International Conference on Smart Computing (SMARTCOMP).

^h^ICST: 2016 International Conference on Selected Topics in Mobile & Wireless Networking (MoWNeT).

^i^FAS: Fertility and Sterility.

^j^ICET: 2021 IEEE 4th International Conference on Electronics Technology.

^k^FII: Frontiers in Immunology.

^l^SSS: *Sensors*.

^m^AJOG: American Journal of Obstetrics & Gynecology.

### Thematic Evolution of the Field

**Figure 5:**
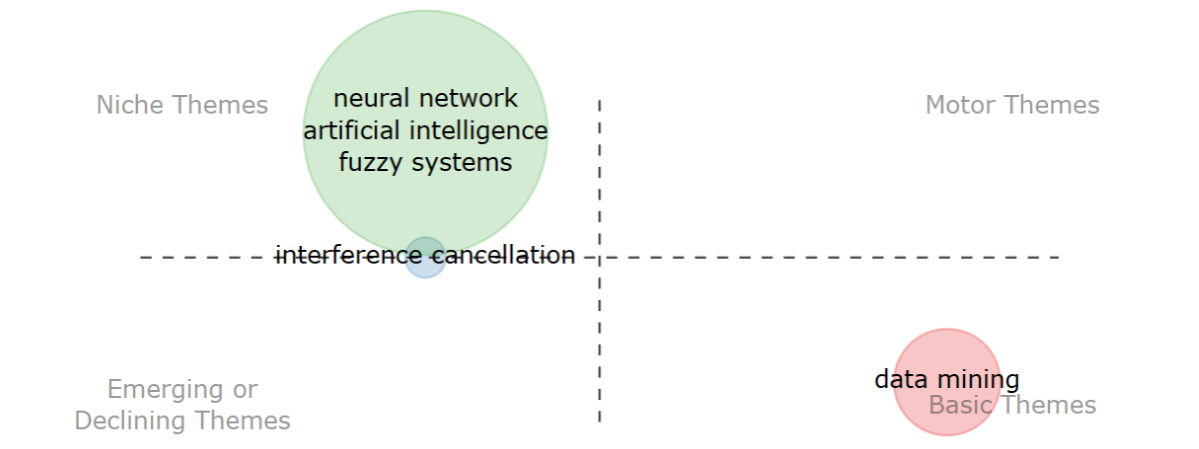
Thematic map for the period 1985-2015.

**Figure 6:**
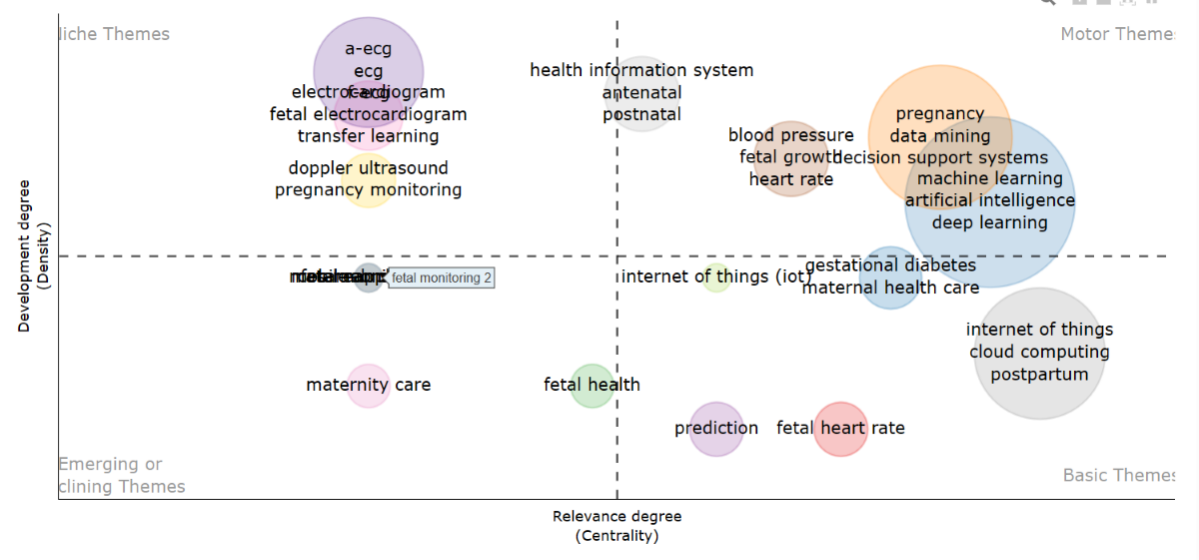
Thematic map for the period 2016-2022. ECG: electrocardiogram.

The first period from 1985 to 2005 and the second period from 2006 to 2015 were combined since the first period had only 1 paper published. The evolution of I4.0 development in maternal health care was thus analyzed across 2 periods, from 1985 to 2015 and from 2016 to 2022. Figures 5 and 6 represent the strategic maps of the main themes and trends for each period based on the authors’ 250 most frequently used words. Table 4 lists the keywords per cluster (I4.0 technologies) by their frequency.

**Table 4:** Cluster keywords and period.

Period and cluster		Keywords
**1985-2015**		
	Neural network	“Neural networks,” “artificial intelligence,” and “fuzzy systems”
	Data mining	“Data mining”
**2016-2022**		
	Machine learning	“Artificial intelligence,” “deep learning,” “neural networks,” “risk prediction,” “telemedicine,” and “digital health”
	Internet of Things	“IoT,” “wearable devices,” “m-healthcare,” and “cloud computing”

The most highly developed theme was neural networks, including AI and fuzzy systems. Artificial neural networks were mostly used as classifiers. Normal, hypertensive, and preeclamptic pregnancies using maternal heart rate variability indexes were explored [38]. Another predominant use case was the prediction of high-risk preterm births using neural networks [31]. AI and fuzzy systems were used to analyze extracted fetal electrocardiogram data [48,49].

Figures 5 and 6 show that there has been a shift in themes focusing on I4.0 from niche themes that are considered of marginal importance in the research field to themes with high density and centrality. From 2016 to 2022, new themes such as deep learning, IoT, and cloud computing were developed. Prior to the 2016-2022 period, several factors hindered the widespread adoption of cloud computing in health care. These included concerns among users regarding data security and privacy, a perceived loss of control over data governance, and a lack of trust in the compliance practices of cloud service providers [50,51]. In 2016-2022, cloud computing was widely accepted despite security concerns and regulations were adopted to address these concerns [52]. Moreira et al [42] and Lopez et al [40] developed an IoT-based system to monitor hypertension in pregnant women, with alerts sent in the event of a change in hypertension. Sarhaddi et al [46] combined the IoT and AI to monitor maternal health during pregnancy and postpartum. Vitals of pregnant women were collected using sensors and further analyzed for anomalies using AI. Mhajna et al [47] developed a self-applied wireless abdominal belt to obtain fetal heart rate and maternal heart rate. Conoscenti et al [53] postulated that connected devices spread sensitive personal patient data and thus privacy can only be guaranteed by the system’s technical design. They proposed a peer-to-peer system, particularly blockchain, to architect privacy-preserving in IoT systems. Fernandez-Carames and Fraga-Lamas [54] used blockchain to ensure, among others, seamless authentication, data privacy security, and robustness against attacks. However, it must be noted that from the identified I4.0 technologies that have impacted health care, as suggested by Paul et al [12], blockchain in maternal health care has not gained widespread adoption in health care; this may be due to technical challenges, regulatory uncertainties, and the lack of interoperability between different blockchain platforms [3].

## Discussion

### Conceptual Framework

**Figure 7:**
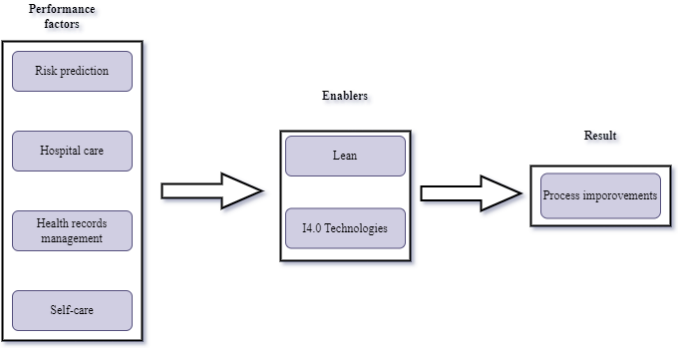
Conceptual framework. I4.0: Industry 4.0.

The World Health Organization recommends that all pregnant women have at least 4 antenatal care (ANC) assessments carried out in the following cycles: 8-12 weeks, 24-36 weeks, 32 weeks, and 36-38 weeks [55]. According to Busumani et al [56], timely ANC booking helps to identify and monitor expecting mothers who might be at risk. Recordkeeping is essential when it comes to monitoring patients’ health. For example, good ANC is crucial as it aims to prevent or detect complications during pregnancy. Pregnant women are encouraged to share information with their health care providers during pregnancy. Sibanda et al [3] posit that 1 area that stands to benefit from adopting manufacturing practices is maternal health care. With many processes in maternal health care, it stands to benefit by adopting Lean practices. According to Ilangakoon et al [57]⁠, Lean practices are tools and techniques used in manufacturing to improve processes. The authors further suggest that Lean tools and techniques, when used in health care (Lean health care), simplify health care processes by adding value and eliminating processes that lead to waste. This section suggests a conceptual framework adapted from Ilangakoon et al [58] and a research agenda. Figure 7 shows the conceptual framework that combines I4.0 technologies and Lean practices in health care as developed from the reviewed literature.

Four factors contributing to the operational performance in maternal health care were identified from the reviewed literature and they are risk prediction, hospital care, health records management, and self-care. These factors must be optimized to streamline services and focus on value-adding services. Therefore, from the literature and the work by Ilangakoon et al [58]⁠, I4.0 technologies and Lean practices were identified as enablers. When performance is optimized, it results in improved patient care, timely disease diagnosis, ease of access to patient records by all stakeholders, patient involvement in their care, and innovative product development.

As noted in Table 5, to enhance operational performance in maternal health care, 4 critical factors from the literature were identified: risk prediction, hospital care, health records management, and self-care. Directly optimizing these factors influences the delivery of value-adding services such as timely treatment, risk analysis, self-monitoring, and reduced hospital wait times. We propose that I4.0 technologies and Lean practices can enable this optimization. For example, AI-powered risk prediction tools facilitate timely disease diagnosis, while secure and accessible electronic health records promote patient involvement in their care. We anticipate enhanced patient outcomes, seamless information sharing among collaborators, and improved treatment plans by effectively using these enablers. Our results highlight the prominence of I4.0 technologies, AI, cloud computing, BDA, and IoT in improving maternal care. As expected, blockchain technology, being relatively new, is less widely adopted within the health care sector at present.

**Table 5:** Performance improvement of maternal processes with I4.0 technologies.

Performance factor	Process improvement	Sources
Risk prediction	Timely treatment and risk analysis may prevent more extended hospitalizations, detailed examinations, and waiting for diagnostics. With AI^a^ and BDA^b^, longer diagnostic processes and corrective treatment plans can be managed more effectively. Doctors can predict risk and find mitigatory measures.	[35,39,41,59]
Hospital care	The waiting time of the patient to receive medical service or preparation when being admitted to the hospital in an emergency is an issue that needs consideration. In the event of a complicated pregnancy, vital parameters of the patient can be sent ahead before admission into a hospital so that precautionary arrangements may be made and waiting time saved. Using the IoT^c^ and the cloud to store sensor data allows vital parameters to be collected and sent before admission.	[14,60,61]
Health records management	During ANC^d^ and postnatal care, different stakeholders play a role in managing a pregnant woman, depending on the state of the pregnancy. In a hierarchical health care system, if a lower-level facility cannot assist, they may refer the pregnant woman to a higher-level facility. However, in some cases, the woman can visit any secondary health care facility by self-referral. Thus, records must be kept for ease of sharing between health facilities and to remove the burden of carrying out tests that would have been done at another facility. This also reduces the time to treatment.	[5,6]
Self-care	Unlike other care processes, ANC is preventive and may be carried out at home or a health care facility accessible to the mother. Frequent consultations are encouraged. However, it can be noted that these consultations might clog the hospital systems and, in some developing countries, care facilities may be further from where the expectant mother is. Systems using the IoT, cloud computing, BDA, and AI have been developed to allow for home-based care. Sensors monitor the woman at home and provide data to caregivers remotely. In the event of an anomaly, alerts are sent. The expectant mother can also self-monitor through smart wearable devices or smartphones.	[7-9]

^a^AI: artificial intelligence.

^b^BDA: big data analytics.

^c^IoT: Internet of Things.

^d^ANC: antenatal care.

### Conclusions

#### Discussion

This paper reviewed the state of the art in the evolution and structure of I4.0 technologies in maternal health care and how they may be used to optimize the operational processes. The study examined the state of research over the period of 1985-2022. We critically evaluated 37 years of I4.0 technologies in maternal health care to identify the main research themes in the field, identify opportunities, and set a research agenda. The study period was divided into 3 periods—1985-2005, 2006-2015, and 2016-2022. For analysis purposes, the first 2 periods were combined. A total of 136 publications during the entire period were identified and reviewed. There was a notable increase in the last period accounting for 121 publications. The research in this period reflects the innovative use of I4.0 technologies. The tools developed help doctors lessen the burden of constantly monitoring patients by providing automated recommendations to patients sent through wearable devices or smartphones. Technologies such as the IoT and cloud computing allow for easy data collection, data sharing among medical practitioners, and remote care of expectant mothers, while AI and BDA allow medical practitioners to gain more insights into patient data.

A noteworthy increase in the amount of research literature can be observed in bibliometric performance. Moreira and Rodrigues are the most prolific authors who have been key in developing I4.0 in maternal health care. Regarding scientific mapping, a conceptual framework was developed from the identified clusters with 4 performance factors suggested—risk prevention, hospital care, health records management, and self-care. Furthermore, how I4.0 technologies can be used to improve operational processes was discussed.

#### Contributions to Theory, Implications for Practice, and Research Agenda

The main contributions of this study were identifying research themes and their evolution, a conceptual framework informed by the literature, and performance factors to optimize operations in maternal health care processes.

Regarding implications for practice, our research has suggested a conceptual framework with 4 performance factors—risk prediction, hospital care, health records management, and self-care, which serve as pillars to optimize operations in maternal health care. This could be useful for health care facilities that want to optimize their operations, those overwhelmed by patients, or those in resource-limited countries with a huge turnover of health care professionals. Moreover, innovative ideas may be adapted to suit their environments from the identified use cases of I4.0 technologies in maternal health care. Further stakeholders such as policy makers, technologists, and health care professionals need to consider the areas that have a potential impact on technological advancement and they are explained in the next section.

#### Telemedicine and Remote Medicine

The IoT significantly expands the potential for remote monitoring and self-care in maternal health, reducing the frequency of hospital visits. Stakeholders must explore how wearables and remote monitoring devices enhance prenatal and postnatal care access, particularly within underserved communities. Key considerations include improving affordability, addressing the digital divide, and providing appropriate patient training to ensure equitable access to these technological advancements.

#### AI, Data Sharing, and Analytics

AI promises to improve predictive modeling to identify high-risk pregnancies at an earlier stage, enabling earlier interventions. Additionally, AI can contribute to developing personalized treatment plans for individual patients. However, addressing the potential for bias within data sets used for these applications is crucial to ensure equitable outcomes for all patients. Secure data-sharing platforms driven by technologies such as blockchain have the potential to facilitate the exchange of maternal health data across institutions while maintaining patient privacy. Big data analysis derived from these data can then inform public health interventions aimed at reducing disparities in maternal health care access and outcomes.

#### I4.0 Technologies and Collaboration

In pursuing effective maternal care practices, effective collaboration models are crucial for developing and implementing technology-driven maternal care solutions. Key considerations should be the best practices in building effective partnerships among all stakeholders, namely policy makers, technologists, and health care professionals.

#### Regulatory Frameworks and Ethics

Interdisciplinary teams should prioritize addressing ethical concerns related to privacy, consent, and equity when deploying new technologies, especially AI use in health care [62]. Considerations should be made on how teams can work together to address the ethical issues regarding privacy, patient consent, and equitable deployment of I4.0 technologies. Furthermore, with a lack of regulatory frameworks for deploying these technologies, stakeholders should proactively address the regulatory complexities.

Regarding theoretical contributions, our bibliometric review helped identify the leading journals for literature and publication, the universities and institutions leading in research, and the most prolific and leading researchers in the field for possible collaboration. The review outlined the research field’s evolution and development and suggested a research agenda. In terms of the latter, a researcher may be able to discover new research avenues in the following sections.

#### Governance Considerations for Implementing I4.0 Technologies in Health Care

Much research focused on the use of AI in maternal health care. However, most of the reviewed literature was conceptual in the form of frameworks, models, and prototypes with no implementations on production systems. This can be attributed to ethical issues in adopting AI in mainstream systems. There is thus scope for further research to identify the challenges and obstacles limiting the implementation of prototypes in an actual medical setting and the development of ethical frameworks. IoT-based systems collect data from various devices (“things”) remotely from expectant mothers to be used in health care facilities to monitor them and, in some cases, made available to research institutions. The issue now is who the owners of the data are. How should that data be used? The ownership of health data is a sensitive issue that warrants further scrutiny.

#### Developing Adoption Frameworks or Models

It can be noted that most of the research to solve challenges in maternal health care is coming from outside Africa, yet the continent has a high maternal mortality rate. However, the developed solutions can be adapted to the African context. Health care processes are many and varied and most are not standardized, which brings challenges in adapting the developed solution to another context. Another issue is the interoperability challenges between disparate systems. There is scope for further research on developing frameworks and models to guide the interoperability of disparate systems. Furthermore, research is needed on how the I4.0 technologies can be applied in maternal health care to optimize processes and integrate them into existing systems.

#### Infrastructure, Privacy, and Security Mechanisms

IoT applications collect data through the use of many sensors, and in most cases, they are used in conjunction with technologies such as cloud computing to store data. Data are analyzed for insights using BDA tools. Adequate infrastructure is required for flexible performance and management of large volumes of data, which is an issue that is critical in the proper performance of IoT applications. Unauthorized access to sensor data in transit or stored on the cloud threatens patient data integrity and this problem needs to be resolved. Blockchain and more specifically, nonfungible tokens have been used to secure and guarantee data ownership. This open research area needs to be explored within the context of maternal health care.

### Limitations

Our study has limitations that deserve acknowledgment. While we are confident that Scopus, a multidisciplinary database publishing peer-reviewed research, provided a valuable foundation for our analysis, future studies could benefit from including additional databases like Web of Science to potentially capture a broader range of relevant publications. Additionally, the scope of explored I4.0 technologies could be broadened. This study focused on 5 key technologies, but future research could investigate the potential applications of 3D printing, augmented reality, virtual reality, digital twins, and cyber-physical systems in improving maternal health care. Finally, while “maternal health” was a broad keyword for this initial investigation, a more refined set of keywords might be necessary in future studies to ensure a comprehensive capture of specific research within the broader field.

## Abbreviations

AI: artificial intelligence
ANC: antenatal care
BDA: big data analytics
I4.0: Industry 4.0
IoT: Internet of Things
NP: number of publications
SDG: Sustainable Development Goal
